# Specific versus Nonspecific Solvent Interactions of a Biomolecule in Water

**DOI:** 10.1021/acs.jpclett.3c01763

**Published:** 2023-11-16

**Authors:** Lanhai He, Lukáš Tomaník, Sebastian Malerz, Florian Trinter, Sebastian Trippel, Michal Belina, Petr Slavíček, Bernd Winter, Jochen Küpper

**Affiliations:** † Center for Free-Electron Laser Science, Deutsches Elektronen-Synchrotron DESY, Notkestraße 85, 22607 Hamburg, Germany; ‡ Institute of Atomic and Molecular Physics, Jilin University, 130012 Changchun, China; ¶ Department of Physical Chemistry, University of Chemistry and Technology, Technická 5, 16628 Prague, Czech Republic; § Molecular Physics, Fritz-Haber-Institut der Max-Planck-Gesellschaft, Faradayweg 4-6, 14195 Berlin, Germany; ∥ Institut für Kernphysik, Goethe-Universität Frankfurt, Max-von-Laue-Straße 1, 60438 Frankfurt am Main, Germany; ⊥ Center for Ultrafast Imaging, Universität Hamburg, Luruper Chaussee 149, 22761 Hamburg, Germany; # Department of Physics, Universität Hamburg, Luruper Chaussee 149, 22761 Hamburg, Germany

## Abstract

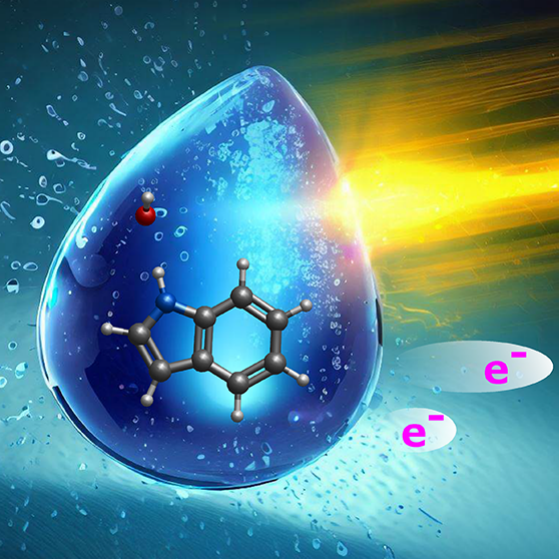

Solvent interactions, particularly hydration, are vital in chemical and biochemical systems. Model systems reveal microscopic details of such interactions. We uncover a specific hydrogen-bonding motif of the biomolecular building block indole (C_8_H_7_N), tryptophan’s chromophore, in water: a strong localized N–H···OH_2_ hydrogen bond, alongside unstructured solvent interactions. This insight is revealed from a combined experimental and theoretical analysis of the electronic structure of indole in aqueous solution. We recorded the complete X-ray photoemission and Auger spectrum of aqueous-phase indole, quantitatively explaining all peaks through *ab initio* modeling. The efficient and accurate technique for modeling valence and core photoemission spectra involves the maximum-overlap method and the nonequilibrium polarizable-continuum model. A two-hole electron-population analysis quantitatively describes the Auger spectra. Core–electron binding energies for nitrogen and carbon highlight the specific interaction with a hydrogen-bonded water molecule at the N–H group and otherwise nonspecific solvent interactions.

Indole (C_8_H_7_N) is a ubiquitous component of peptides and proteins, as it is the side-chain chromophore of the essential amino acid tryptophan. Indole has various signaling functions,^1−3^ and most relevant for the present work, it starts to absorb strongly below 300 nm, with the main bands around 287 and 217 nm, both corresponding to π → π* transitions.^4,5^ The influence of solvation on indole’s electronic structure has long been debated,^6^ in particular with respect to its two lowest-energy ^1^*L*_*a*_ and ^1^*L*_*b*_ electronically excited singlet states.^7−9^ Microsolvation experiments with one or a few water molecules attached have shed some light onto this topic.^10−12^ One of many roles of tryptophan is the radiation protection of nucleic acids, e.g., as a near-UV-absorbing building block of the eumelanin polymer.^13,14^ Indole became one of the widely studied model systems to understand the photochemistry of heteroaromatic systems, in particular, the role of the πσ* states.^1^ The excited states and photodynamics of gaseous indole were studied by a large variety of techniques, including high-resolution spectroscopy,^8,9,15^ pump–probe laser spectroscopy,^16,17^ ion imaging,^18^ and *ab initio* calculations.^19^ Radiation below 300 nm has the potential to cause pyrimidine dimerization, e.g., leading to melanoma.^20^ The most damaging is radiation around 250 nm, corresponding to a minimum of the indole absorption spectrum. While the nucleic acids in solution dominantly suffer radiation damage indirectly, via reactions with OH radicals and hydrated or prehydrated electrons formed upon irradiation of water,^21,22^ they can be also ionized directly.^23,24^ Then, charge migration and transfer take place, and indole can be one of the sinks for the positive holes formed.^25,26^ Redox properties of indole were studied for decades by using kinetic techniques. There were multiple experimental and theoretical studies of indole’s photoelectron spectrum in the gas phase.^27−31^ Recently, the electronic structure of indole was investigated in a combined experimental and theoretical study,^31^ using tunable X-ray radiation and *ab initio* electron propagation and density functional theory. So far, the complete electronic structure of indole in the solvent environment, and particularly in water, has not been reported. However, the resonant two-photon ionization (R2PI) spectrum of aqueous-phase indole^32^ and the valence photoelectron spectrum of aqueous-phase tryptophan,^33^ with its indole side chain, provided information on indole’s valence electronic structure.

Here, we used soft X-rays in conjunction with a liquid microjet to record the full photoemission spectrum, including valence, core, and Auger electrons of indole in an aqueous solution. Previous liquid-jet photoemission studies have provided accurate insight into the electronic structure of various molecular species, including nucleic acids, organic chromophores, anions, cations, and transition metal complexes.^34−36^ Assisted by electronic-structure calculations, we assign the major experimental features, and we specifically disentangle nonspecific bonding interactions, brought about by the long-range solvent polarization, and the specific effects, related to the granularity of the solvent environment, e.g., hydrogen bonding with the nearest solvent molecules. From a computational aspect, we demonstrate the wide applicability of the so-called maximum-overlap method,^37^ enabling us to model the ionized states with the standard ground-state quantum chemical methods.

## Experimental Methods

The X-ray photoelectron-spectroscopy experiments were carried out at the P04 beamline of the PETRA III synchrotron-radiation facility at DESY.^38^ Experimental details of the liquid microjet and photoelectron spectrometer as part of the “Electronic structure of Aqueous Solutions and Interfaces” (EASI) setup were described elsewhere.^39^ Briefly, solutions were prepared by mixing highly demineralized water (18.2 MΩ·cm^–1^) and 17 mM indole (Sigma-Aldrich, >99%, used without further purification). Sodium chloride (50 mM) was added to minimize the streaming potential caused by electrokinetic charging.^40−46^

A liquid jet of ∼28 μm diameter, with a velocity of ∼60 m/s from a fused-silica nozzle, is generated using a high-performance liquid chromatography (HPLC) solvent delivery pump with a constant flow rate and backing pressure. The liquid jet was captured/dumped in a vacuum by using a cryopump. The indole–water solution temperature was estimated to be in the range of 279–283 K in the laminar part, which typically exists for 10 mm after the jet injection from the capillary into vacuum.^40^

The synchrotron light beam, with a photon energy of 600 eV and a focal size of 180 μm in the horizontal direction (parallel to the liquid jet) and 35 μm in the vertical direction (perpendicular to the liquid jet), intersected the jet perpendicular to the flow of the solution. The small focal size allowed for matching the spatial overlap with the liquid jet, thereby keeping the signal contributions from the ionization of gas-phase water molecules surrounding the jet low. The excitation was carried out with circularly polarized light, and a backward-scattering electron-detection geometry, corresponding to an angle of 130° with respect to the light propagation direction, i.e., near the magic angle, as detailed elsewhere.^39^ The emitted electrons passed from the main interaction chamber, operated at 10^–4^ mbar, through an 800 μm diameter orifice to the differentially pumped detector chamber, operated at ∼10^–8^ mbar, which housed a hemispherical electron energy analyzer equipped with a microchannel-plate detector. The small jet diameter, in conjunction with the small distance of 800 μm between the liquid jet and the orifice, assured that a significant fraction of detected electrons did not inelastically scatter with water gas-phase molecules near the jet surface.^40,41^ The energy resolution of the P04 beamline was better than 250 meV. The energy resolution of the hemispherical analyzer was better than 200 meV. Therefore, the total energy resolution was better than 320 meV. Tuning the detecting kinetic-energy range of the hemispherical electron energy analyzer, we experimentally obtained the photoelectron spectra (PE spectra) from the aqueous-phase indole’s valence band as well as from core ionization of the nitrogen and carbon 1s orbitals, including the Auger electrons.

## Theoretical Methods

Photoelectron spectra were modeled within the nuclear-ensemble method, which can be viewed as a particular realization of the reflection principle, i.e., by projecting the nuclear density in the electronic ground state onto the ionized state and further onto the spectrum of ionization energies.^47−49^ The ground-state density of indole (without water molecules) was estimated within the path-integral molecular-dynamics method, accelerated with the quantum thermostat based on the generalized Langevin equation^50^ (PI+GLE method). In this way, the delocalization due to the nuclear quantum effects was taken into account. The simulation was done at the BLYP/6-31G* level of electronic-structure theory, representing an efficient combination for a large number of calculations during the MD simulation. A time step of 0.5 fs was used, and four replicas were propagated. The total duration of the simulation was 23 ps with the first 5 ps used for equilibration. In total, 100 indole geometries were used for the subsequent calculations. PI+GLE simulations were performed using our in-house code ABIN^51^ connected to the Terachem software (version 1.93).^52,53^

Core-level ionization spectra were calculated at the MP2 level of theory with the cc-pCVTZ basis set (designed specifically for core states) for carbon and nitrogen atoms and the corresponding cc-pVTZ basis set for hydrogen atoms. For modeling the core-ionized states, we have used the maximum-overlap method (MOM)^37^ as implemented in the Q-Chem software (version 4.3).^54^ The aqueous solution was mimicked by the nonequilibrium polarizable-continuum model with integral equation formalism (IEF-PCM)^55^ with the atomic radii from universal force field (UFF)^56^ and the electrostatic scaling factor by which the sphere radius is multiplied α = 1.1. Note that IEF-PCM does not include nonelectrostatic contributions and is, therefore, an approximative approach to the description of solvation. In total, core-ionization energies from 100 sampled geometries were obtained for every atom, i.e., one nitrogen and eight carbon atoms. The PE spectra of the aqueous-phase indole were constructed as a sum of Gaussian functions centered at the respective calculated value of the ionization energies for each geometry. The standard-deviation parameter for every single Gaussian function was set to 0.32 eV, obtained using the additional broadening model,^57^ and corresponds to the additional broadening arising from different configurations of solvating water molecules that were not explicitly included in our calculations. The model connects the spectral width to reorganization energy using an approximative “universal” relaxation frequency of water after the solute’s ionization. The model required the reorganization energy of water molecules solvating indole, which we calculated as a difference between the energies calculated with the equilibrium and nonequilibrium versions of the PCM solvation model for the optimized indole geometry. The gas-phase PE spectra were generated with the same procedure except for the standard-deviation parameter that was set according to Silverman’s rule of thumb.^48,58^

The valence-ionization spectrum was calculated using the long-range-corrected Perdew–Burke–Ernzerhof functional (LC-ωPBE)^59^ with the aug-cc-pVDZ basis set. The range-separation parameter ω was optimized to the value of 0.3 *a*_0_^–1^ on the set of 100 indole geometries using the ionization-potential theorem, i.e., minimizing the difference between ionization energies obtained as the energy of the HOMO and as the difference between electronic energies of the ground and ionized states.^60,61^ Using the optimized parameter ω, we subsequently calculated the first two ionization energies for each geometry with the MOM approach. The photoelectron spectrum was generated by the same procedure described above. We have tested and confirmed the robustness of our ionization-energy calculations; the detailed results are presented in the Supporting Information.

Furthermore, we explored the influence of involving explicit water molecules in our calculations on the resulting ionization energies. To do so, we (i) calculated ionization energies of solvated clusters containing the indole molecule with one to three explicit water molecules, (ii) executed the molecular-dynamics simulation (distinctive from PI+GLE described above) of indole involving explicit water molecules, and then calculated the valence-ionization spectrum for snapshots of the MD simulation. In (i), the microhydrated clusters were optimized at the MP2/cc-pVTZ level of theory, and core- and valence-ionization energies for the minimal geometries were calculated using the same approach as described above. In (ii), the molecular-dynamics simulation was performed for a droplet of indole in water, using the QM/MM approach with the quantum part containing the indole molecule and the MM part consisting of 500 water molecules. The QM part was described at the BLYP/6-31g* level with Grimme’s dispersion correction D2,^62^ the MM part was described with the TIP3P water model.^63,64^ Calculations were performed at the standard temperature of 298.15 K during the whole simulation by the Nosé–Hoover thermostat. The QM/MM simulation was performed using our code ABIN connected to the Terachem software (version 1.93), the initial arrangement of water molecules was obtained using the Packmol code.^65^ The total duration of the simulation was 25 ps with time steps of 0.5 fs. From the last 20 ps, 100 geometries were sampled with equidistant steps, i.e., every 200 fs. We have then extracted the coordinates of the indole molecule without water molecules and with its closest 20 water molecules, respectively. Those structures were then used to calculate the valence-ionization spectrum following the procedure described above (including PCM) to inspect the influence of explicit water molecules on the calculated valence-ionization energies.

The onset of the Auger-electron spectra was calculated as the difference between the core-ionized and double-valence-ionized electronic energies calculated at the MP2 level with the cc-pCVTZ basis set for N and C atoms and the cc-pVTZ basis set for H atoms. The core-ionized states were described with the MOM method. The solvation was described by the nonequilibrium PCM model. The Auger spectra were modeled by two different approaches for a single geometry optimized at the MP2/cc-pVTZ level. In the first approach, we evaluated the onsets of the spectra within the MOM method with correlated wave functions, and then we modeled the higher transitions with a simpler *ab initio* approach based on the complete active space configuration interaction (CAS-CI) wave function expansion. The Auger intensities were estimated within the qualitative approximate scheme based on the Mulliken population analysis.^66^ In this method, relative transition rates are approximated by using atomic populations of valence orbitals of particular final states on core-ionized atoms. The valence orbitals are constructed by the CAS-CI method and the cc-pVTZ-f basis on the neutral-ground-state wave function. The kinetic energies of the Auger electrons *E*_*i*_ were evaluated as
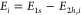
where *E*_1*s*_ is the energy of the core-ionized state and *E*_2*h*,*i*_ is the energy of the final two-hole state. The spectrum was then shifted to match the onset described above. The second approach for the calculation of Auger energies and intensities utilized the Feshbach–Fano approach implemented in Q-Chem 6.0.^67^ The initial core-ionized states are described using the fc-CVS-EOM-CCSD (frozen-core core–valence separated equation-of-motion coupled-cluster singles and doubles) method, while the final doubly ionized states are described by the EOM-DIP-CCSD (equation-of-motion double-ionization potential coupled-cluster singles and doubles) method. The uncontracted version of the aug-cc-pVDZ basis set was used, according to a recent publication.^68^ Due to the high computational cost of an EOM-DIP-CCSD method, only approximately 50 lowest-lying doubly ionized singlet and triplet final states are covered by the approach. The Auger energies and transition rates were calculated in the gas phase and then shifted by the solvent shift calculated at the same level of theory using the PCM solvation model and MOM method for obtaining core-ionized states. In both approaches, we started with core-ionized states localized on different atoms: eight carbons and one nitrogen in total. The line spectra consisting of energies and intensities from the contributions of final states were broadened with a Gaussian distribution with a standard deviation of 2 eV to reproduce the broadening observed in the experiment. The calculations were performed in the development-version TeraChem software (version 1.9)^52,53^ and Q-CHEM (version 6.0).^69^

## Valence Photoemission

**Figure 1:**
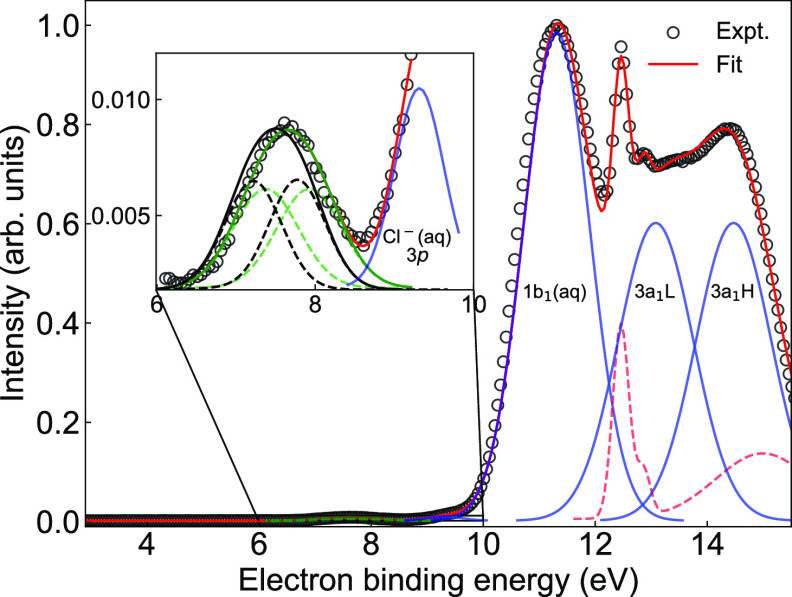
Experimental valence photoelectron spectrum (gray empty circles) from a 17 mM indole aqueous solution containing 50 mM NaCl. The solid red line represents the overall fit. The contributions from the 1*b*_1_ and 3*a*_1_ molecular orbitals of liquid water, with the 3*a*_1_ components 3*a*_1_*L* and 3*a*_1_*H*, and Cl^–^(3p) of sodium chloride background, are indicated in the figure with blue solid lines. A dashed red line indicates the contributions from gaseous H_2_O. The inset shows the details of the binding-energy region between 6 and 10 eV with the indole photoemission signal. The experimental data were either fitted with one Gaussian (green solid line) or with two Gaussian functions (green dashed lines) representing the signals from the HOMO and HOMO–1 orbitals of aqueous-phase indole; see text for details. The black solid line represents the calculated valence spectrum of aqueous-phase indole, and the black dashed lines represent the calculated components from HOMO and HOMO–1.

The PE spectrum of the valence band (VB) of an indole-water solution, measured at a photon energy of 600 eV, is shown in Figure 1. The raw spectrum was energy-calibrated against the liquid water 1*b*_1_ peak at 11.33 eV.^46,70−75^ In the present study, no low-energy cutoff measurements from an electrically biased liquid jet have been performed. This is the reason we cannot apply the more robust method for the determination of absolute electron binding energies, as recently reported.^75^ We expect the uncertainty of the reported energies to be on the order of 100–300 meV. Photoemission signals (gray empty circles) are fitted with multiple Gaussian functions, representing the contributions from the involved atomic and molecular orbitals, and the red solid line represents the overall fit.

By comparing the valence-band PE spectrum of the indole-water solution to that of a 50 mM NaCl solution in water, we identified the emerging peak that exclusively corresponds to aqueous-phase indole, shown in the inset of Figure 1 with fitted contributions (green lines). We used two approaches for fitting the measurement, i.e., either one Gaussian or two Gaussian functions. The binding energy extracted from the single-Gaussian fit is 7.65(1) eV with a full width at half-maximum (fwhm) of 1.18 eV. The spectral width reflects, in a nontrivial way, the reorganization energy of the solute upon single ionization. However, the observed 1.18 eV value is significantly larger than to be expected for aromatic molecules of similar size in solution.^35^ Therefore, this immediately indicates that this band corresponds to the ionization from multiple orbitals; i.e., the peak spans at least two close-lying molecular orbitals. This tentative conclusion is supported by the corresponding gas-phase data, showing the HOMO and HOMO–1 orbitals to be separated by merely 0.45 eV; see Table 1. It is further supported by the previously reported valence photoelectron spectrum of aqueous-phase tryptophan^33^ as well as by our *ab initio* calculations, as we discuss below. The energies of these two orbitals (green dashed lines in the inset of Figure 1) have been extracted, from a fit using two Gaussian functions, to be 7.38 and 7.93 eV with a fwhm of 0.97 eV; the difference between the HOMO and HOMO–1 energies was fixed to 0.55 eV based on our calculations; see details below, and the amplitudes of the two transitions were set to be identical, assuming the same cross sections for ionization from HOMO and HOMO–1 orbitals of indole. The extracted energies are in good agreement with the published photoelectron-spectroscopy data for aqueous-phase tryptophan,^33^ with HOMO and HOMO–1 energies of 7.3 and 8.0 eV, respectively.

**Table 1:** Vertical Ionization Energies (VIE) of Aqueous-Phase and Gaseous Indole, in Units of eV

	Exp._*aq*_	Calc._*aq*_	Exp._*g*_^a^	Calc._*g*_
HOMO	7.38	7.22	7.90	7.86
HOMO–1	7.93	7.77	8.35	8.34

a VIE reported for indole in the gas phase in ref (31).

Upon transfer of the molecule from the gas phase into an aqueous solution, the ionization energy is expected to be shifted due to the polarizable environment.^76^ The extent of the solvent shift is controlled by the relative stabilization of the initial neutral ground state and the final radical-cation state in water. A typical shift observed for aromatic molecules of a size similar to indole is on the order of 1 eV.^35^

Our calculated valence-ionization energy in the gas phase agrees excellently with the experimental data of Plekan et al.^31^ with a discrepancy in binding energies of less than 0.05 eV; see Table 1. As for the calculated aqueous-phase valence spectrum including HOMO and HOMO–1 contributions, Figure 1 (black solid line in the inset) has its maximum at 7.50 eV, differing from our experimental aqueous-phase data by 0.15 eV. This is a very good agreement; the remaining discrepancy is to be attributed to the treatment of solvation. The observed shift in the present case is on average ∼0.47 eV for the HOMO and HOMO–1 orbitals; see Table 1. The exact value is slightly dependent on the fitting procedure. However, it is surprisingly small, suggesting competing specific and nonspecific solvent effects. The calculated spectrum exhibits a spectral shape, which is in good agreement with the present experiment. Note that the calculated spectra were generated within the cluster-continuum model; i.e., the solvent was described as a polarizable continuum while, simultaneously, the 20 nearest water molecules were included in the calculations. If these explicit molecules were excluded, the maximum of the calculated photoelectron peak would be shifted to 7.38 eV, compared to 7.50 eV. This identified certain specific solvent effects, manifested by a difference of 0.12 eV. These specific solvent effects counter, to some extent, the nonspecific effects and are responsible for the relatively small solvent shift observed in the experiment compared to similar molecules.

**Figure 2:**
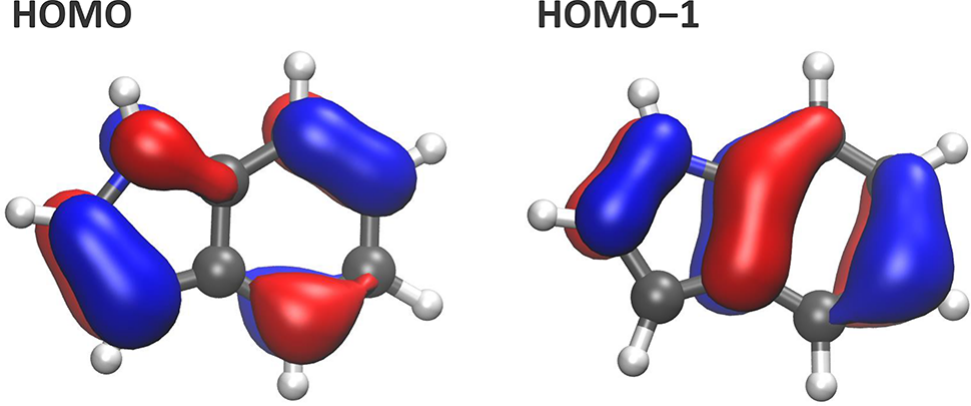
Calculated highest occupied molecular orbital (HOMO) and second-highest occupied molecular orbital (HOMO–1) of aqueous-phase indole. Both orbitals are π-characterized and are delocalized over the molecule.

The calculations reveal that the aqueous-phase valence band actually consists of two transitions corresponding to two different orbitals, both of them of π character; see Figure 2. The difference in the peak positions based on our calculations is estimated to be 0.55 eV. This is somewhat larger than the 0.45 eV splitting for the gas-phase experiment, which is reproduced very well by our *ab initio* calculations. However, the discrepancy is not significant when we consider the inaccuracies in the calculations and the errors introduced by the fitting procedure for the experimental data.

**Figure 3:**
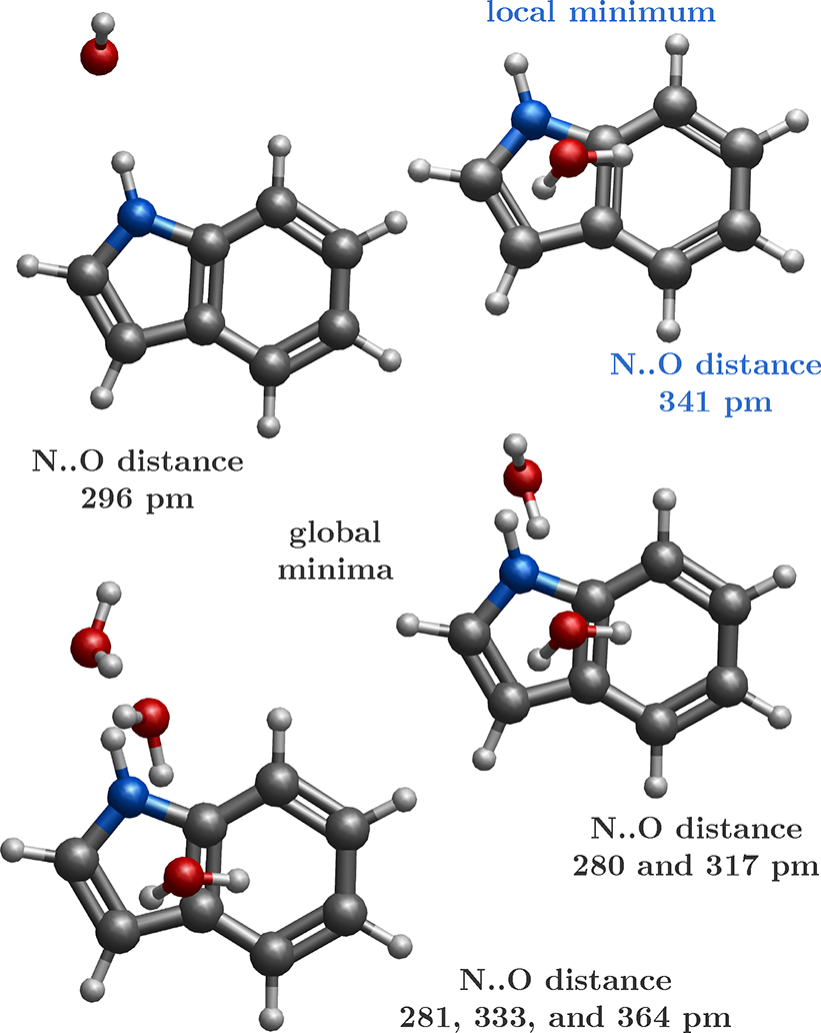
Optimized structures of gas-phase indole–water clusters containing one, two, and three water molecules. At least one water molecule forms a hydrogen bond with the N–H group, while others are pointing toward the π ring.

Let us now focus on the interplay between specific and nonspecific solvent interactions. An interesting insight is brought about by the inspection of the indole–water dimer complex in the gas phase. The global minimum of this complex corresponds to the hydrogen-bonded structure via the N–H·O contact,^10^ see also Figure 3. This complex has a calculated vertical ionization energy (VIE) of 7.51 eV and an adiabatic ionization energy (AIE) of 7.27 eV. These calculations can be compared with the available experimental data, where R2PI experiments provided AIEs of the bare indole molecule as well as the cluster of indole with one water molecule of 7.76 and 7.34 eV, respectively.^77^ The energetic drop of about 0.4 eV in experimental AIE when adding the first water molecule is also observed in our calculations of VIEs, which drop from 7.87 to 7.51 eV; see Table 3. Water molecules can, however, also bind to the π system of the indole molecule; see Figure 3. The hydrogen-bonded complex is energetically preferred by 0.1 eV according to our calculations. The VIE for this complex is calculated to be 0.6 eV larger while the adiabatic energy is only 0.3 eV larger than for the π-system-bound structure. Thus, the reorganization energy is larger for the dimer with the water molecule bound to the π system of indole than that for the hydrogen-bonded system. At the same time, the optimized structure of the oxidized indole-water complex is very different from the neutral structure in this case.

In liquid water, the water molecules are delocalized over the entire indole molecule. One of the water units is certainly located in the hydrogen-bonded position as it is documented by the core-level ionization spectrum; see subsection Core-Electron Binding Energies below. Most other water molecules are placed above or below the π ring of the indole system. In those positions, the water molecules will need to dramatically reorganize upon ionization, which will lead to pronounced reorganization energy and a decreasing value of the Franck–Condon overlap for the states energetically close to the AIE. The water molecules surrounding the π system also contribute to the increase of the VIE, counterbalancing the VIE decrease by long-range polarization. As a result, the solvent shift is smaller than expected from the dielectric theory or from our experience with organic molecules of a similar size.

## Core-Level Binding Energies

**Figure 4:**
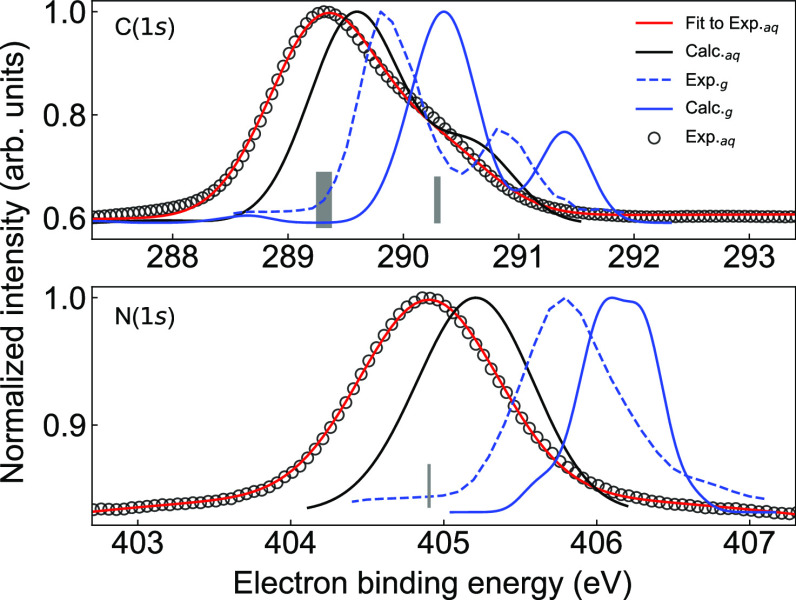
C(1s) (upper panel) and N(1s) (lower panel) photoelectron spectra of indole. Gray empty circles represent the experimental data, and red solid lines represent the overall fit for aqueous-phase indole. The light-gray bars indicate the central positions of the individual Gaussian functions used to fit. The black and blue solid lines represent the simulated spectra for aqueous-phase indole and gas-phase indole, respectively. The blue dashed lines are experimental data reported for indole in the gas phase.^31^

The carbon and nitrogen 1s core-level PE spectra of aqueous-phase indole, measured at 600 eV photon energy, are shown in Figure 4 together with a fit of multiple Gaussian functions. Spectra are displayed on an electron binding energy (BE) axis, and the raw spectra were background-corrected by subtracting second-order polynomials to account for the contributions of electrons scattered in the solution. As in the case of the valence spectra, BE calibration was performed against the liquid water 1*b*_1_ (HOMO) peak at 11.33 eV, and we expect 100–300 meV uncertainty of the energies reported here.

The C(1s) PE spectrum of gaseous indole showed two distinct features, one centered at 289.89 eV arising from the six carbon atoms not bound to nitrogen and one centered at 290.86 eV arising from the two carbon atoms directly bound to nitrogen,^31^ based on gas-phase results. For aqueous-phase indole, these two features largely merge, and we fitted the spectrum simultaneously with two groups of Gaussian functions (6 + 2). Thus, the characteristic fitted parameters were mainly two independent peak positions. The fwhm for each Gaussian function was fit to be 1.12 eV, which is fully consistent with the values typically found for the photoionization of neutral molecules of a similar size in solution. For the N(1s) PE spectrum, resulting from the single nitrogen atom in indole, one Gaussian function centered at 404.90 eV with a fwhm of 1.06 eV was used to fit the spectrum. Compared to the reported gas-phase data,^31^ we obtained solvent-induced shifts of ∼0.57 eV for C(1s) and ∼0.92 eV for N(1s).

The N(1s) and C(1s) BEs for aqueous-phase and gaseous indole from both the experiment and our calculations are shown in Table 2. Note that the calculated values for C(1s) were obtained by fitting the simulated C(1s) core-level PE spectrum with the same fitting procedure used for the experimental spectrum. The fwhm of each Gaussian function in the fit for the C(1s) simulated spectrum is 0.92 eV. The simulated N(1s) spectrum for aqueous-phase indole is centered at 405.28 eV with a fwhm of 0.58 eV. The calculated PE spectra for the N(1s) and C(1s) electrons from gas-phase and aqueous-phase indole were shifted with respect to the experiment by ∼0.3–0.4 eV, which is quite acceptable for these large absolute energies. More importantly, the calculations reproduce well the shapes of the spectra in both the gas and liquid phases, including the broadening upon solvation and the solvent shift; see Figure 4. Even the C(1s) spectrum corresponding to convoluted signals from eight different carbon-atom sites in the molecule is described well for both aqueous- and gas-phase indole.

**Table 2:** C(1s) and N(1s) Experimental and Calculated Binding Energies (eV) for Aqueous-Phase and Gas-Phase Indole

Peak	Exp._aq_	Calc._aq_	Exp._g_^a^	Calc._g_
C^1–6^	289.31	289.59	289.89	290.34
C^7–8^	290.29	290.58	290.86	291.39
N^1^	404.90	405.28	405.82	406.15

a Gas-phase ionization energies reported in ref (31).

## Solvation Structure

While valence electrons are to a large extent delocalized, core electrons are localized near one of the nuclei. X-ray PES is thus suited for investigating the local or nearest-neighbor hydrogen-bonding structures by analyzing core-level chemical and solvent shifts as well as peak profiles arising from the intermolecular interaction of indole with surrounding water molecules.^78^

The solvent-induced shifts for the valence, C(1s), and N(1s) signals are significantly different. This is indicative of the specific solvation structure near the ionized molecules. Energy shifts are approximately 0.47 eV for the valence ionization, 0.57 eV for the C(1s) ionization, and 0.92 eV for the N(1s) ionization.

When we examine the geometries of the microhydrated indole, Figure 3, it is clear that one closest water molecule tends to approach the nitrogen atom to form a direct N–H···O hydrogen bond, reflecting the structure of the gas-phase indole–water dimer.^10^ This leads to a larger solvent stabilization of the core-ionized nitrogen atom as the water molecule’s dipole is pointing at indole’s N; i.e., the water is oriented toward N–H with its oxygen atom. On the other hand, core-ionized carbons are destabilized by the arrangement of the other water molecules. Those waters are hydrogen-oriented toward the indole ring. Therefore, the positively charged core holes on carbon atoms created by the ionization interact with partial positive charges of water’s hydrogen atoms.

This interpretation is further supported by calculations showing how calculated core- and valence-ionization energies of indole change upon adding explicit solvent molecules and introducing the polarizable continuum representing bulk water; see Table 3. We observed that the addition of a single water molecule coordinated to the nitrogen atom causes a large decrease in the N(1s) binding energy, and this holds even when the system is placed into a dielectric environment. The effect of this hydrogen-bonded water molecule on the valence and C(1s)-electron energies is much smaller. This is related to the delocalized character of the valence-electron hole and to the diffuse hydrogen-bond arrangements around the core-ionized carbon atoms, respectively.

**Table 3:** Calculated Ionization Energies (eV) of Gas-Phase and Aqueous-Phase Indole Containing 0–3 Explicit Water Molecules^a^

	Gas-phase cluster					Solvated cluster				
*n*_*water*_	HOMO	HOMO–1	N(1s)	C^1–6^(1s)	C^7–8^(1s)	HOMO	HOMO–1	N(1s)	C^1–6^(1s)	C^7–8^(1s)
0	7.87	8.29	406.24	290.15	291.24	7.13	7.57	405.28	289.39	290.43
1	7.51	7.94	405.55	289.82	290.82	7.03	7.48	404.95	289.32	290.30
2	8.02	8.42	405.99	289.72	291.28	7.22	7.64	405.15	289.46	290.45
3	7.93	8.33	405.74	290.21	291.17	7.18	7.60	404.98	289.43	290.40

a “Solvated cluster” refers to a system placed into a dielectric environment to mimic non-specific solvation effects. For clarity, C(1s) ionization energies are averaged for six (1–6) and two (7–8) carbon atoms, which exhibit very similar energies.

## Auger-Electron Spectra

**Figure 5:**
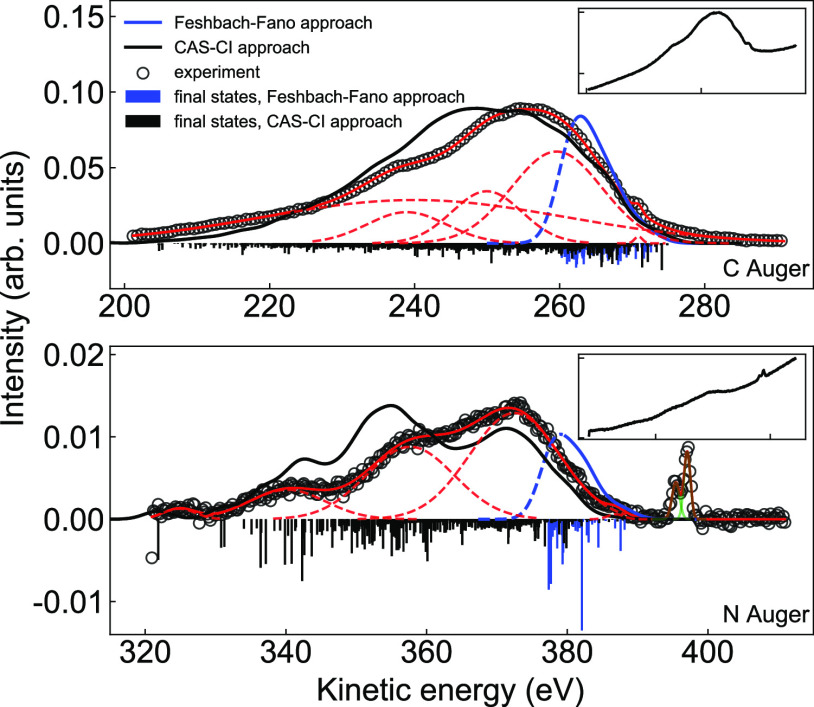
C-Auger and N-Auger spectra (black circles) of aqueous-phase indole after the fitted background subtraction. Red solid lines represent the overall fit and the red dashed lines are the individual fitted Gaussian functions. The black solid lines represent the simulated carbon/nitrogen 1s Auger spectra via the CAS-CI approach, and the blue solid lines represent simulated carbon/nitrogen Auger spectra via the Feshbach–Fano approach. Dashed blue lines describe a part of the modeled spectra, for which a significant contribution of higher-lying doubly ionized states is expected, but is absent due to the method limitations. Contributions from the ionization of Cl(2p) are included in the fit and are shown as green solid lines. The downward-facing sticks schematically represent the population of the involved two-hole states. The insets show the raw experimental Auger spectra. A global fit using second-order polynomials for the contributions of the scattered-electron background, with a highly asymmetric spectral structure, was applied.

Measured Auger-electron spectra following N(1s) and C(1s) core-level ionization of aqueous-phase indole at 600 eV photon energy are shown in Figure 5 (top and bottom, empty circles), respectively. Here, we have subtracted a linear background from the as-measured spectra shown in the respective insets. Both spectra are broad, approximately 60–70 eV wide. Similar to that for gaseous indole,^31^ the broadening includes the finite line width of the X-ray PE spectrum, the vibrational distributions of the core-hole ground and final states, the core-hole lifetime, the analyzer energy resolution, and the contributions from the solvation for aqueous-phase indole due to the conformational distribution of solvating molecules. The spectra were fitted with five Gaussian functions for both C-Auger and N-Auger data, corresponding to a minimal number of functions, yielding a reasonable total fit of the experiment. Note though that these Gaussian functions have no physical meaning, and only the high-KE part of the spectrum will be analyzed here. The applied photon energy is well above the respective core-level energies such that participator and spectator Auger channels do not need to be considered. The sharp doublet peak near 396 eV kinetic energy (KE), next to the N-Auger spectrum, arises from Cl^–^(2p) core-level ionization; as explained in the Methods section, 50 mM NaCl has been added to the indole aqueous solution to counteract electrokinetic charging.

The high-kinetic-energy onsets of the N(1s) and C(1s) Auger-electron spectra are roughly 388 and 274 eV, respectively, in accordance with the calculated values as shown in Table 4. The KE was calibrated with reference to the energies of the C(1s) and N(1s) photoelectron peaks, as explained above. The measured Auger spectra have been interpreted with the help of *ab initio* modeling using the approach based on Mulliken population analysis. In addition, we used the more sophisticated yet more costly method based on the Feshbach–Fano approach. In either case, Auger peaks are found to be formed by transitions involving a large number of final states, and the peaks can thus not be interpreted as a result of a single decay channel. The total calculated Auger-electron spectra using Mulliken population analysis, shown as solid black lines in Figure 5, are seen to reasonably reproduce both experimental spectra, even exhibiting the experimentally observed substructure. The solid blue lines in Figure 5 show the calculated Auger spectra by using the Feshbach–Fano approach. This allowed us to directly estimate both nitrogen and carbon leading Auger-electron contributions, including the respective high-energy edges. However, for a molecule of the size of indole (and larger), only a limited number of final doubly ionized states can be captured due to the computational cost, not allowing to model the whole Auger spectra. This is reflected in the dashed blue curve, below which spectra can no longer be reliably calculated due to the missing calculated final states. We can conclude that a much simpler approach based on Mulliken population analysis reproduces the whole spectrum, and this method is sufficient for the present study.

**Table 4:** Summary of the Measured and Calculated BEs (eV) for Valence, N(1s), and C(1s) Ionization and the KE Onsets of the Auger Electrons, for Aqueous-Phase Indole

	Exp. fitting		Simulations	
	BE	KE onset	BE	KE onset
HOMO	7.38	–	7.22	–
HOMO–1	7.93	–	7.77	–
N(1s)	404.90	–	405.28	–
C^1–6^(1s)	289.31	–	289.59	–
C^7–8^(1s)	290.29	–	290.58	–
N Auger	–	387.53	–	387.02
C Auger	–	274.31	–	272.26

The electron holes of the final state correspond to the ejected π electrons of the indole ring; see the HOMO and HOMO–1 orbitals in Figure 2. Calculated values for gas-phase indole are 384.74 and 269.79 eV for N- and C-Auger electrons, respectively, in good agreement with recently published experimental data.^31^ The calculated solvent shifts for nitrogen and carbon Auger energies are 2.28 and 2.47 eV, respectively, confirming the higher N(1s) core-ionized solvent stabilization relative to the C(1s) core-ionized state.

In conclusion, we have provided the first full photoemission spectrum of indole in aqueous solution by measuring the valence and core-level photoelectron and Auger spectra following ionization with 600 eV synchrotron radiation. Experimental spectra are interpreted with the help of high-level *ab initio* calculations. All characteristic peaks of aqueous-phase indole were assigned, and the explicit and global solvent-induced energy shifts were extracted and supported by the calculations.

The lowest-binding-energy valence photoelectron peaks correspond to the ionization of the HOMO and HOMO–1 electrons with binding energies of 7.38 and 7.93 eV, respectively. The observed solvent-induced shifts were relatively small in comparison with those of other neutral molecules of a similar size. This is due to delocalized valence electrons and competing specific and nonspecific solvation effects.

Indole, in the gas phase, is known to form strong hydrogen-bonded complexes with water with its N–H group serving as the hydrogen-bond donor.^10^ Our results demonstrate that this motif is also dominant in aqueous solution. Specifically, disentangling the solvent-induced shifts, which are specific extensions of the general chemical shift, in the core-ionization spectra enabled us to elucidate the solvent structure around the indole molecule. The core-level binding energies for nitrogen and carbon 1s electrons clearly indicated the presence of specific solvent effects due to a strong hydrogen bonding to nitrogen together with further nonspecific effects due to solvent polarization. On the one hand, there is a strong directed and specific hydrogen bonding N–H···OH_2_, while on the other hand, there are unstructured interactions of the water solvent with the overall molecular structure.

Furthermore, we reported and interpreted the Auger spectra, which exhibit larger solvent shifts in comparison to the direct photoelectrons. These Auger-electron signals are brought about by many final dicationic states. A computational technique based on electron-population analysis was demonstrated as an efficient tool for modeling Auger spectra, aiding in the further analysis of molecules in complex environments using X-ray photoemission spectroscopy.

Overall, our detailed investigation of the photoemission spectrum of indole in water provided a clear and refined view of the solvation of indole, specifically for its different moieties, including a surprising highly specific single-solvent molecule-binding motif combined with further unspecific solvent interactions. From the perspective of computational chemistry, our work demonstrated the wide applicability of the maximum-overlap method, enabling us to model the ionic states through standard ground-state quantum chemical methods, combined with the nonequilibrium dielectric modeling of the environment.

## Data Availability

The data of relevance to this study have been deposited on Zenodo at DOI: 10.5281/zenodo.6519525. The ABIN code for molecular dynamics v.1.1-alpha was used, available at https://github.com/PHOTOX/ABIN. The Packmol code v.18.169 was used, available at https://github.com/m3g/packmol. Terachem v.1.93 was used, available at https://store.petachem.com. Q-Chem v.4.3 and 6.0 was used, available at https://www.q-chem.com.

## Supporting Information Available

Calculated gas-phase photoelectron spectrum of indole, details on modeling Auger spectra, and robustness test of ionization-energy calculations (PDF)
