# Supplementary material for: Specific versus Nonspecific Solvent Interactions of a Biomolecule in Water
Source: J Phys Chem Lett. 2023 Nov 16;14(46):10499–508. doi: 10.1021/acs.jpclett.3c01763 (PMC10683073; doi:10.1021/acs.jpclett.3c01763)
Supplement: Supplementary file 1 — jz3c01763_si_001.pdf [file jz3c01763_si_001.pdf]

Supplementary Information: Specific versus Non-Specific Solvent Interactions of a Biomolecule in Water

Lanhai He,[†,‡,@] Lukáš Tomaník,[¶,@] Sebastian Malerz,[§] Florian Trinter,[§,‖] Sebastian Trippel,[†,⊥] Michal Belina,[¶] Petr Slavíček,[*,¶] Bernd Winter,[*,§] and Jochen Küpper[*,†,⊥,#]

†*Center for Free-Electron Laser Science CFEL, Deutsches Elektronen-Synchrotron DESY, Notkestraße 85, 22607 Hamburg, Germany*

‡*Institute of Atomic and Molecular Physics, Jilin University, 130012 Changchun, China*

¶*Department of Physical Chemistry, University of Chemistry and Technology, Technická 5, 16628 Prague, Czech Republic*

§*Molecular Physics, Fritz-Haber-Institut der Max-Planck-Gesellschaft, Faradayweg 4-6, 14195 Berlin, Germany*

‖*Institut für Kernphysik, Goethe-Universität Frankfurt, Max-von-Laue-Straße 1, 60438 Frankfurt am Main, Germany*

⊥*Center for Ultrafast Imaging, Universität Hamburg, Luruper Chaussee 149, 22761 Hamburg, Germany*

#*Department of Physics, Universität Hamburg, Luruper Chaussee 149, 22761 Hamburg, Germany*

@*These authors contributed equally.*

E-mail: Petr.Slavicek@vscht.cz; winter@fhi-berlin.mpg.de; jochen.kuepper@cfel.de

Valence photoemission

Figure S1 shows our simulated valence-band spectrum for gaseous indole in comparison with experimental photoemission results.[1] The calculated valence-ionization energies are in excellent agreement with the experimental data for both the overall shape and the peak positions. The discrepancy in binding energies is less than 0.05 eV.

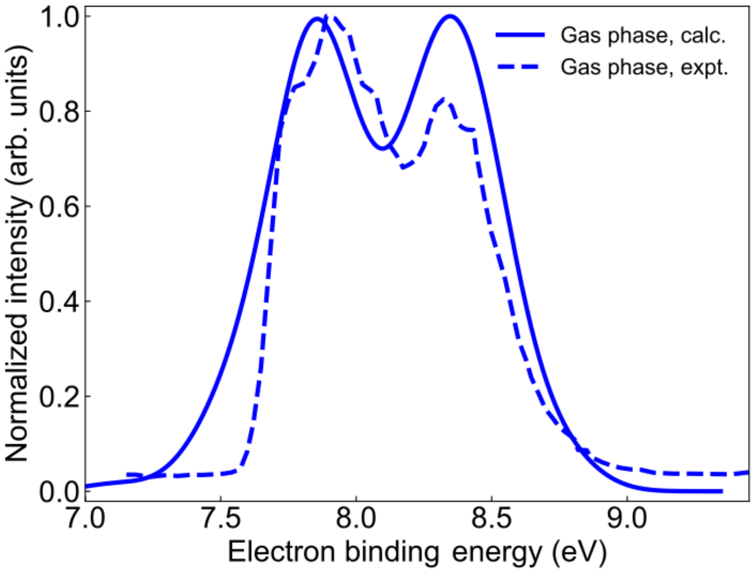

Figure S1: Valence photoemission spectrum of gas-phase indole. The blue solid line represents our simulated spectrum and the blue dashed line the experimental data reported elsewhere.[1]

Auger electrons

Figure S2 shows our computed Auger spectra following the ionization of aqueous-phase indole from both the carbon and nitrogen 1s orbitals, based on *ab initio* modeling. The experimental spectra, shown in Figure 5 of the main text, are reasonably reproduced for both cases, including the experimentally observed substructure. The underlying spectra of two-hole states show that the peaks in the Auger spectra are composed of large numbers of final states, *i.e.*, the peaks must not be interpreted as single decay channels.

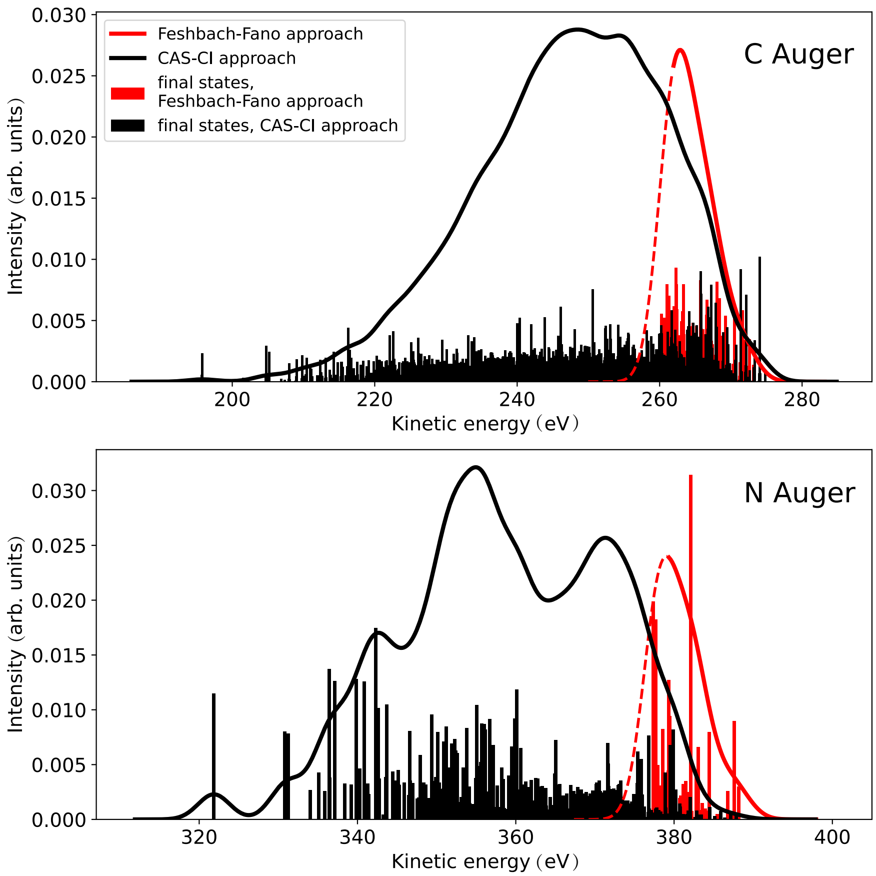

Figure S2: Theoretical (black solid line) carbon (upper panel) and nitrogen (lower panel) 1s Auger spectrum modeled by the approach based on Mulliken population analysis (CAS-CI final states). The positions and intensities ($\times 3$ for better visibility) of the particular 2h states are indicated by black vertical bars. The red solid line depictures the modeled carbon (upper panel) and nitrogen (lower panel) 1s Auger spectrum using the Feshbach-Fano approach. The positions and intensities ($\times 3$ for better visibility) of particular 2h states are indicated by red vertical bars. The red dashed line describes a part of the modeled spectra, for which a significant contribution of higher-lying doubly ionized states is expected, but is absent due to the method limitations. The dashed region was set to go from the lowest energies to the energy obtained by adding 2 eV to the lowest-lying transition. 2 eV represent the standard deviation used for spectra broadening.

Solvation structure

Our conclusions about the solvation structure of indole in the main text are in agreement with our molecular dynamics. The performed QM/MM dynamics of indole surrounded by 500 water molecules exhibited at least one water molecule to be hydrogen-bonded to the N-H group of indole in the vast majority of time frames.

Robustness of ionization-energy calculations

Here, we present results of various tests on the robustness of our computational approach for ionization energies.

Number of time frames from molecular dynamics

Table S1 demonstrates the dependence of calculated ionization energies on the number of time frames used. All the calculated energies are clearly converged with 100 geometries used.

Table S1: Dependence of calculated ionization energies (eV) on the number of time frames used.

	10 frames	50 frames	100 frames
HOMO	7.23	7.21	7.22
HOMO−1	7.74	7.76	7.77
Nitrogen	405.30	405.21	405.21
Carbon	289.55	289.57	289.60

Number of explicit water molecules

For the valence-ionization calculations, the combined explicit/implicit model of solvation was used. We explicitly included 20 surrounding water molecules in the calculations to include short-range solute-solvent specific interactions. In Table S2, we show the results calculated on the subset of five geometries sampled from our molecular dynamics (its details are described in the main text). Even though 20 explicit water molecules might not provide a full

convergence, the number was used to compromise accuracy and computational capabilities.

Table S2: Dependence of calculated HOMO ionization energies (eV) on different numbers of explicit solvating water molecules.

n_{water}	0	5	10	20	25
HOMO	7.38	7.53	7.52	7.49	7.47

Cavity size in polarizable-continuum model

The polarizable-continuum model (PCM) of implicit solvation contains an important parameter – the electrostatic scaling factor, by which the sphere radius is multiplied (α). We have tested the robustness of our approach regarding α on the subset of five geometries sampled from our molecular dynamics (its details are described in the main text). Table S3 shows the HOMO ionization energies calculated for α in the range 1.0–1.2. The differences are rather small, therefore, $\alpha = 1.1$ used in our work is justified.

Table S3: Dependence of calculated HOMO ionization energies (eV) on the electrostatic scaling factor, by which the sphere radius is multiplied (α).

	$\alpha = 1.0$	$\alpha = 1.1$	$\alpha = 1.2$
HOMO	7.13	7.19	7.22

References

(1) Plekan, O. et al. Experimental and theoretical photoemission study of indole and its derivatives in the gas phase. *J. Phys. Chem. A* **2020**, *124*, 4115–4127.
